# The Relationship Between Bisphosphonate Adherence and Fracture: Is It the Behavior or the Medication? Results From the Placebo Arm of the Fracture Intervention Trial

**DOI:** 10.1002/jbmr.274

**Published:** 2010-10-11

**Authors:** Jeffrey R Curtis, Elizabeth Delzell, Lang Chen, Dennis Black, Kristine Ensrud, Suzanne Judd, Monika M Safford, Ann V Schwartz, Douglas C Bauer

**Affiliations:** 1 Division of Clinical Immunology and Rheumatology, University of Alabama at BirminghamBirmingham, AL, USA; 2 Department of Epidemiology, University of Alabama at BirminghamBirmingham, AL, USA; 3 Department of Epidemiology and Biostatistics, University of California at San FranciscoSan Francisco, CA, USA; 4 Department of Medicine and Epidemiology, University of MinnesotaMinneapolis, MN, USA; 5 Department of Medicine, Minneapolis Veterans Administration Medical CenterMinneapolis, MN, USA; 6 Department of Biostatistics, University of Alabama at BirminghamBirmingham, AL. USA; 7 Division of Preventive Medicine, University of Alabama at BirminghamBirmingham, AL, USA; 8 Departments of Medicine and Epidemiology and Biostatistics, University of California at San FranciscoSan Francisco, CA, USA

**Keywords:** COMPLIANCE, ADHERENCE, HIP FRACTURE, VERTEBRAL FRACTURE, ALENDRONATE, BISPHOSPHONATE

## Abstract

Medication compliance may be a surrogate for factors that improve health outcomes such as fractures. Little is known about the size of this potential “healthy adherer” effect. We evaluated the hypothesis that compliance with placebo is associated inversely with bone loss and fractures among women participating in the Fracture Intervention Trial (FIT). Compliance with placebo and alendronate was evaluated using daily medication diaries. Women were defined as having high compliance if they took 80% or more of dispensed study medication. Change in bone mineral density (BMD) was assessed using mixed models comparing women with high versus lower compliance with placebo. Cox proportional-hazards models analyzed the association between placebo compliance and various types of fractures. Among 3169 women randomized to placebo, 82% had high compliance. Compared with women with lower placebo compliance, bone loss at the total hip was lower in compliant placebo-treated women (−0.43%/year versus −0.58%/year, *p* = .04). Among placebo-treated women, there were 46 hip, 110 wrist, 77 clinical vertebral, and 492 total clinical fractures. Compared with women with lower placebo compliance, women with high placebo compliance had a nonsignificant reduced risk for hip fracture [adjusted hazard ratio (HR) = 0.67, 95% confidence interval (CI) 0.30–1.45]. This trend was not observed for other fractures. Medication compliance may be a proxy for factors that confers benefit on reducing hip fracture (but not other types of fractures) independent of the effect of the medication itself. Nonrandomized studies of interventions designed to maintain or improve bone density and/or hip fracture may need to consider medication compliance as a confounder to better estimate true intervention effects. © 2011 American Society for Bone and Mineral Research.

## Introduction

Several studies have reported a strong inverse relation between high compliance with oral bisphosphonates and fracture risk.(1–5) A recent review has summarized many of these studies and shown that long-term compliance with oral osteoporosis medications generally is low and that women with high compliance who took at least 80% of prescribed medication had a substantially lower risk for fracture than less compliant women.(6) However, concerns have been raised that medication compliance itself is associated with factors that may have a favorable impact on outcomes. This finding, sometimes called the “healthy adherer effect,” is a potential source of confounding that is not always accounted for in analyses. Data supporting the importance of the healthy adherer effect come from several sources, including a meta-analysis of eight clinical trials showing that high compliance with placebo was associated with a 44% lower rate of death.(7) High compliance also has been associated with health-seeking behavior, use of preventive services (eg, immunization and cancer screening tests), and a lower rate of traumatic accidents.(8,9) However, the existence and magnitude of the healthy adherer effect has not been examined previously with respect to fracture outcomes.

The Fracture Intervention Trial (FIT) was a trial that was conducted in 11 US communities in the 1990 s, included over 6000 women, and tested the efficacy of alendronate in improving bone mineral density (BMD) and reducing fracture risk.(10–12) We used data from the FIT to evaluate the hypothesis that high compliance with placebo was associated with lower rates of bone loss and fracture. We also evaluated changes in compliance following a fracture, hypothesizing that women would be more likely to be noncompliant following a fracture.

## Methods

### Study population

The FIT was a placebo-controlled, double-blind trial of 6469 women randomized to alendronate versus placebo.(10,11) The FIT-I and FIT-II cohorts included women with low bone mass (defined as a *T*-score of less than −1.6 at the femoral neck) with and without existing vertebral fracture at baseline, respectively. Data from both groups of women were pooled for this analysis given comparable compliance to study medication in the two cohorts. Daily calcium intake was estimated by food-frequency questionnaire, and participants in both groups who had calcium intakes of less than 1000 mg were given a daily supplement providing 500 mg of elemental calcium (as the carbonate salt) and 250 IU of cholecalciferol (vitamin D). About 82% of participants received the supplement at the randomization visit.

### Ascertainment of compliance and fracture outcomes

Compliance in FIT was evaluated using daily patient diaries and pill counts returned at annual study visits, which had excellent agreement with one another. Although compliance therefore was defined as a continuous variable ranging from 0% to 100%, women were defined as having high compliance in this analysis if they took 80% or more of dispensed medication, following prior conventions.(6) Fractures were confirmed centrally through review of medical records, as per the FIT protocol.

### Statistical analysis

Descriptive statistics were used to summarize demographics, comorbidities, and BMD data comparing women with high compliance versus others; for purposes of this analysis, all women who did not meet criteria for high compliance were categorized as having lower compliance (< 80%). Compliance was measured in a time-varying manner and measured as average compliance since the beginning of the study. Compliance with placebo was the primary independent variable of interest; outcomes associated with compliance with alendronate also were evaluated for comparative purposes. Cox proportional-hazards models were used to estimate the association between high compliance and hip, clinical vertebral, and wrist fractures. Change in BMD, reported as annualized percent change, was assessed between study annual visits and was analyzed in relation to compliance during this same interval using mixed models. Each patient could contribute multiple observations to the BMD analysis. Factors hypothesized to potentially affect the association between compliance and fractures (eg, age, fracture history, self-reported health status) and baseline BMD were adjusted for in both the survival models and the mixed models.

To better understand the dynamic nature of compliance in the setting of an acute fracture, compliance was reported among patients with hip, clinical vertebral, and wrist fracture, comparing compliance before fracture (ie, from the beginning of the study until the fracture date) with compliance after fracture (ie, from the fracture date until the end of the study). Agreement between pre- and postfracture compliance was quantified using Cohen's kappa statistic with 95% confidence intervals (CIs). All analyses were conducted using SAS 9.2 (SAS Institute, Cary, NC, USA).

## Results

**Fig. 1:**
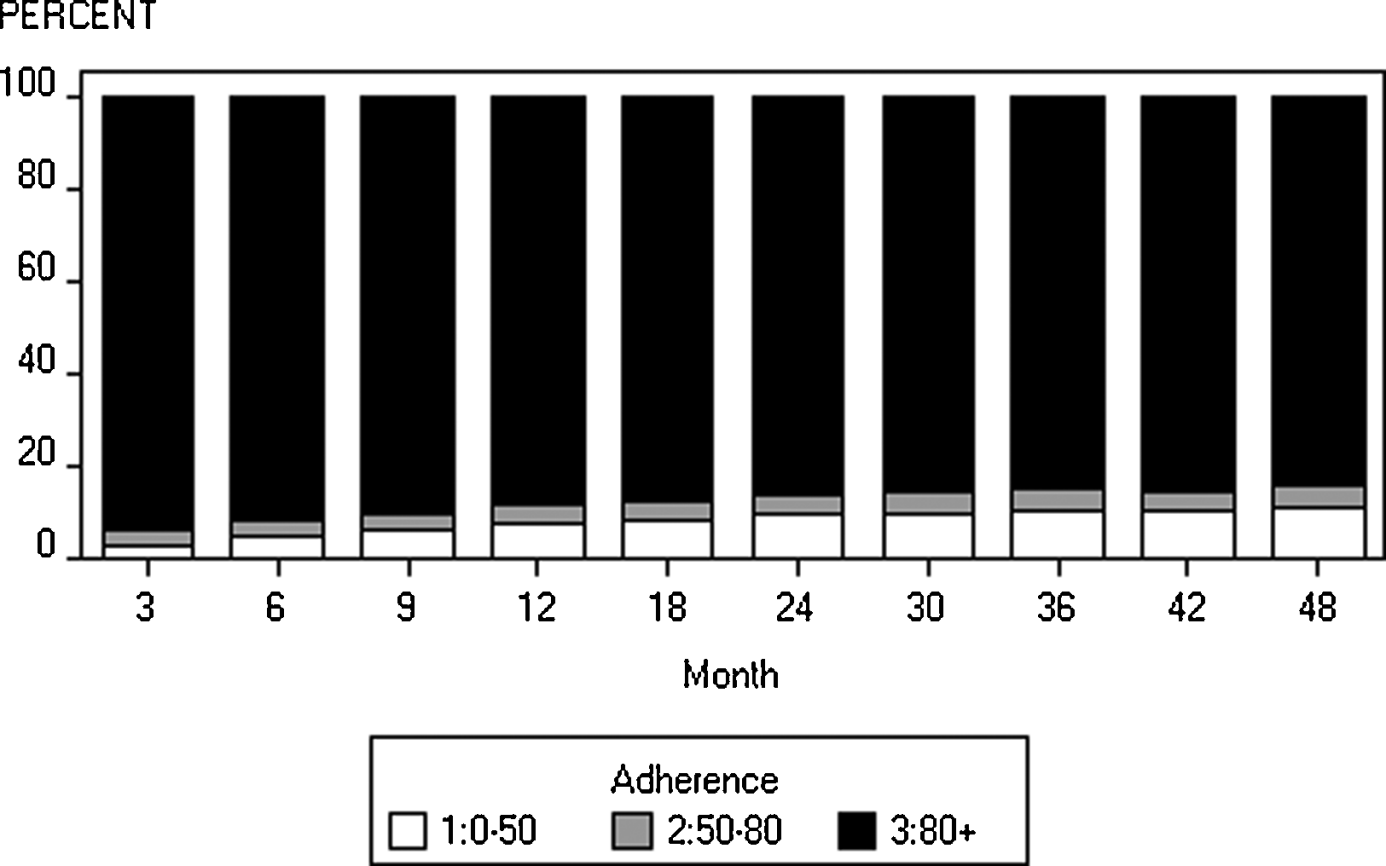
Compliance over the course of FIT. Data shown are the percent of women compliant at each time point.

A total of 3169 women participating in FIT were randomized to placebo. Compliance with the placebo study medication was high over the course of FIT, with 80% to 85% of women having high compliance over the 4 years of the study (Fig. 1). Stratifying these women into high versus lower compliance at the end of FIT, Table 1 describes their characteristics. Women with lower compliance were more likely than highly compliant women to have fair/poor self-reported health and to be current or former smokers.

**Table 1:** Baseline Characteristics of FIT Participants Randomized to Placebo (*n* = 3169)

Variable	High compliance^a^	Lower compliance^a^	*p* Value
Age, %			.49
< 65 years	51.7%	51.0%	
65–74 years	29.8%	28.1%	
75–81 years	18.5%	20.8%	
Mean (SD) age in years	68.2 (6.10)	68.6 (6.2)	.12
Femoral neck, SDs below peak, %			.90
>2.5	42.7%	42.5%	
2.0–2.5	26.1%	27.1%	
1.5–2.0	31.1%	30.4%	
Mean (SD) BMD in g/cm^2^			
Femoral neck	0.58 (0.06)	0.58 (0.07)	.26
Posteroanterior spine	0.83 (0.14)	0.82 (0.13)	.54
History of fractures ≥ 45 years, %	41.9%	43.5%	.49
Vertebral fractures at baseline, %	9.0%	9.9%	.53
Body mass index, mean (SD), kg/m^2^	25.2 (4.1)	25.4 (4.3)	.26
Self-rated health status, %			< .001
Very good/excellent	66.0%	54.5%	
Good	29.6%	36.4%	
Fair/poor	4.5%	9.1%	
Baseline height, mean (SD), cm	1601 (61)	1599 (62)	.64
Dietary calcium intake, mean (SD), mg/d	629 (395)	649 (399)	.77
Smoking, %			.03
Current	10.6%	13.3%	
Former	34.0%	37.2%	
Never	55.4%	49.5%	

*Note:* For this table, compliance was assessed at the end of the study. All other compliance analyses were time-varying. Data were analyzed using chi-square tests for nominal groups and *t* tests for continuous data.

a High compliance defined as 80% or greater; lower compliance defined as less than 80%.

The association between compliance with placebo or compliance with alendronate and change in BMD is shown in Table 2. As shown, women with high compliance with alendronate had a significantly greater increase in BMD at all sites than those with lower compliance with alendronate or placebo-treated women. Women with high compliance with placebo had significantly less BMD loss at the total hip than those with lower compliance with placebo. A similar pattern was observed at the femoral neck but not the lumbar spine.

**Table 2:** Annualized Percent Change in BMD of High and Lower Compliance With Placebo and Alendronate

	Placebo		Alendronate	
		
	Lower compliance	High compliance	Lower compliance	High compliance
Total hip	−0.58^a^,^b^	−0.43^a^	−0.30^a^	0.99
Femoral neck	−0.30^a^,^c^	−0.16^a^	−0.05^a^	1.15
Spine	0.49^a^,^c^	0.48^a^	0.79^a^	2.33

*Note:* Results are adjusted for age, baseline BMD, height, BMI, self-reported health, smoking status, calcium intake, calcium supplement use, having a broken bone after age 45, and having a vertebral fracture at baseline.

a *p* < .0001 compared with alendronate high-compliance group.

^ab^*p* = .04 compared with placebo high-compliance group.

^bc^*p* = NS compared with placebo high-compliance group.

Table 3 shows the adjusted association between compliance with placebo and fracture. Women with high compliance with placebo had proportionately fewer hip fractures than those with lower compliance with placebo. The adjusted rate of hip fracture among women with high placebo compliance was 33% lower than among women with lower placebo compliance, but there were few events, and the results did not reach statistical significance. There was no suggestion of an association between high compliance with placebo and a reduced risk for any other type of fracture. Comparing women with lower versus high compliance with alendronate, there was an approximately 50% lower risk for hip and clinical vertebral fracture among women with high compliance with alendronate than among those with lower compliance with alendronate.

**Table 3:** Risk of Hip, Clinical Vertebral, Wrist, and All Clinical Fractures Comparing High Versus Lower Compliance With Placebo and Alendronate

	Placebo		Alendronate	
		
Fracture type	Lower compliance	High compliance	Lower compliance	High compliance
Hip, *n*	8	38	10	20
Crude rate^a^	5.0	3.6	6.3	1.9
Crude HR	1.0 (referent)	0.67 (0.3–1.45)	1.0 (referent)	0.30 (0.14–0.63)
Adjusted^b^ HR	1.0 (referent)	0.67 (0.30–1.45)	1.0 (referent)	0.46 (0.19–1.10)
Clinical vertebral, *n*	10	67	11	31
Crude rate^a^	6.3	6.4	6.9	2.9
Crude HR	1.0 (referent)	0.99 (0.51–1.94)	1.0 (referent)	0.43 (0.22–0.87)
Adjusted^b^ HR	1.0 (referent)	1.05 (0.53–2.06)	1.0 (referent)	0.51 (0.24–1.09)
Wrist, *n*	11	99	14	91
Crude rate^a^	7.0	9.5	8.6	8.7
Crude HR	1.0 (referent)	1.34 (0.72–2.50)	1.0 (referent)	0.94 (0.53–1.65)
Adjusted^b^ HR	1.0 (referent)	1.18 (0.63–2.23)	1.0 (referent)	1.05 (0.57–1.93)
Any clinical fracture, *n*	57	435	64	349
Crude rate^a^	39.0	44.5	43.2	35.1
Crude HR	1.0 (referent)	1.11 (0.84–1.47)	1.0 (referent)	0.80 (0.61–1.04)
Adjusted^b^ HR	1.0 (referent)	1.06 (0.80–1.41)	1.0 (referent)	0.87 (0.65–1.15)

HR = hazard ratio.

a Per 1000 person-years.

b Adjusted for age, baseline BMD, height, BMI, self-reported health, smoking status, dietary calcium intake, calcium/vitamin D supplement provided by study, having a broken bone after age 45, and having a vertebral fracture at baseline.

Table 4 shows the adjusted risk of fracture comparing alendronate versus placebo among those with both lower and high compliance. Among women with lower compliance with placebo or alendronate, there were no significant differences between the two groups in the rates of any fracture type. In contrast, among women with high alendronate compliance, there was an adjusted and significant 45% lower risk for hip fracture, a 59% lower risk for clinical vertebral fracture, and a 20% lower risk for all clinical fractures than among women with high placebo compliance.

**Table 4:** Risk of Hip, Clinical Vertebral, Wrist, and All Clinical Fractures Comparing Lower Compliance With Placebo Versus Alendronate and High Compliance With Placebo Versus Alendronate

	Lower compliance		High compliance	
		
Fracture type	Placebo	Alendronate	Placebo	Aendronate
Hip, *n*	8	10	38	20
Crude rate^a^	5.0	6.3	3.6	1.9
Crude HR	1.0 (referent)	1.26 (0.50–3.18)	1.0 (referent)	0.52 (0.30–0.90)
Adjusted^b^ HR	1.0 (referent)	0.86 (0.31–2.37)	1.0 (referent)	0.55 (0.32–0.95)
Clinical vertebral, *n*	10	11	67	31
Crude rate^a^	6.3	6.9	6.4	2.9
Crude HR	1.0 (referent)	1.11 (0.47–2.60)	1.0 (referent)	0.46 (0.30–0.70)
Adjusted^b^ HR	1.0 (referent)	0.87 (0.35–2.14)	1.0 (referent)	0.41 (0.26–0.65)
Wrist, *n*	11	14	99	91
Crude rate^a^	7.0	8.6	9.5	8.7
Crude HR	1.0 (referent)	1.27 (0.57–2.79)	1.0 (referent)	0.92 (0.69–1.22)
Adjusted^b^ HR	1.0 (referent)	1.03 (0.45–2.33)	1.0 (referent)	0.92 (0.68–1.23)
Any clinical fracture, *n*	57	64	435	349
Crude rate^a^	39.0	43.2	44.5	35.1
Crude HR	1.0 (referent)	1.11 (0.78–1.58)	1.0 (referent)	0.79 (0.69–0.91)
Adjusted^b^ HR	1.0 (referent)	0.97 (0.67–1.41)	1.0 (referent)	0.80 (0.69–0.92)

HR = hazard ratio.

a Compliance measured in a time-varying way.

b Adjusted for age, baseline BMD, height, BMI, self-reported health, smoking status, dietary calcium intake, calcium/vitamin D supplement provided by study, having a broken bone after age 45, and having a vertebral fracture at baseline.

Compliance with study medication (placebo or alendronate) before and after hip, clinical vertebral, and wrist fracture among patients who fractured during the course of FIT is shown in Table 5. Although most women had high compliance both before and after fracture, women were more likely to change from having high compliance before fracture to lower compliance after fracture. For hip fractures, for example, 12 women who had high compliance prior to the hip fracture had lower compliance following the fracture. No women with lower compliance prior to hip fracture became highly compliant after fracture. Agreement between compliance before and after fracture for each of the three fracture types was good, with kappas in the 0.65 to 0.73 range.

**Table 5:** Comparison of Compliance With Study Medication (Alendronate or Placebo) Measured Before and After Fracture

Fracture type	High compliance before and after fracture	Lower compliance before and after fracture	High compliance before, lower compliance after fracture	Lower compliance before, high compliance after fracture	Agreement^a^ between high compliance at time of fracture versus end of study
Hip	53	11	12	0	0.66 (0.47–0.85)
Clinical vertebral	90	17	10	2	0.65 (0.49–0.81)
Wrist	182	21	10	2	0.73 (0.60–0.86)

*Note:* High compliance was 80% or greater; lower compliance was less than 80%.

a Reported as kappa (95% CI); kappas between 0.60 and 0.80 are generally considered as “good agreement” (Altman et al., 1991).(13)

## Discussion

Among women participating in FIT who were randomized to placebo, we did not find significant associations between compliance with placebo and fractures. However, we found that high compliance with placebo was associated with reduced total-hip bone loss, and a similar trend was observed for changes in femoral neck BMD. Furthermore, compared with women with lower compliance with placebo, the risk of hip fracture was 33% lower among women with high compliance with placebo; this relationship did not reach statistical significance and was not observed for any other clinical fracture type. These findings suggest that the effect of compliance with placebo on hip BMD, and perhaps hip fracture risk, was not attributable to confounding by the known fracture risk factors collected in FIT. In total, our findings provide some support for the existence of the healthy adherer effect in this population and suggest that medication compliance may be a proxy for behaviors and/or other factors that confer hip BMD (and possibly hip fracture) benefit independent of the effect of the medication.

The possible protective effect on hip fracture may be mediated at least in part by changes in BMD, recognizing that the correlation between change in BMD and fracture benefit is only modest.(14) Comparing women with high versus lower compliance with placebo, the annualized difference in change in hip BMD between the two groups was −0.15%. To provide some context for this difference, these changes in BMD are comparable with differences in BMD among depressed women compared with those without depressive symptoms(15) and with the magnitude of BMD loss among women using selective serotonin reuptake inhibitors (SSRIs) compared with nonusers.(16)

In analyses that evaluated the dynamic nature of compliance, we observed that compliance varied before and after fractures. At least in a randomized, controlled trial (RCT), the occurrence of a clinical fracture would appear to affect subsequent adherence with study medication, at least for some patients. This finding also has been reported in observational data from a nonclinical trial population.(1) These results support the conclusion that analyzing compliance in a manner that ignores the time-dependent nature of adherence before and after fractures may yield biased results.

If the healthy adherer effect is generalizable to a nonclinical trial setting, our findings may have implications for future comparative effectiveness research (CER) of osteoporosis medications, at least for hip fracture outcomes. Analyses studying the relative effectiveness of medications that require intravenous administration (eg, zoledronic acid and denosumab) will need to carefully consider how to choose the most valid comparator groups. For example, patients receiving parenteral osteoporosis agents given by a health care provider consist of a mix of patients who would have variable degrees of compliance with oral bisphosphonates. Comparing these patients with patients who are highly compliant with oral bisphosphonates could yield biased results because the compliant oral bisphosphonate users may have better outcomes (including lower hip fracture risk) in part owing to the healthy adherer effect. In contrast, a comparator group of patients starting oral bisphosphonates, without considering whether they remained compliant, would be biased given their lesser exposure to bisphosphonates. These studies could, for example, consider compliance with other medications to at least partially address these concerns. Future work evaluating healthy behaviors and factors that are associated with compliance behavior are necessary to ensure unbiased results from observational comparative effectiveness analyses.

Although our results suggested a trend toward reduced hip fracture risk associated with placebo compliance, we did not observe similar trends for other fracture types, such as wrist or clinical vertebral fractures. Risk factors for wrist fractures are different from those for hip fractures,(17) and wrist fractures generally occur in younger, healthier, and more active women. Factors associated with medication adherence may be more relevant for older, frailer patients at risk for hip fracture. Given that our crude and fully adjusted results were similar for each analysis, it would seem that the risk factors controlled for within this analysis are not important confounders of fracture risk associated with medication adherence. Furthermore, studies that show a fracture benefit associated with medication compliance may not be confounded if fracture types other than hip fractures are being evaluated.

The strengths of our study include a large population of women with median follow-up of more than 4 years. Fracture outcomes were adjudicated through medical record review, and longitudinal BMD data were available over the course of FIT. Compliance was assessed with high precision through the use of both patient diaries and pill counts. Despite these strengths, our results must be interpreted in light of some limitations. Modest numbers of outcome events and generally high compliance within FIT required dichotomizing compliance and resulted in wide confidence intervals for some of our results. Additionally, FIT was a clinical trial of women who were enrolled to study fracture outcomes, and thus these results may not be generalizable to other settings and patient populations. Last, some factors that may affect fracture risk were not collected in FIT, such as exercise, falls, depression, comorbidities, and measures of frailty. The association between compliance and smoking and poorer health status reported in Table 1 suggest the possibility that these two factors may be proxies for one or more of unmeasured confounders.

Another potentially important factor that was not systematically measured in FIT was 25-hydroxyvitamin D. An ancillary study to FIT that sampled 1000 women at baseline showed that only a small proportion (2.3%) were deficient in vitamin D [25(OH)D ≤ 10 ng/mL) at baseline, and the response to alendronate was not affected by baseline 25(OH)D status.(18) However, it is unknown whether low vitamin D at baseline or throughout the study might be associated with low compliance and could have affected for our results.

In conclusion, based on these data from a randomized, controlled trial, we found small but significant differences in the change in hip BMD between women with high versus low compliance with placebo. However, perhaps most important, studies reporting fracture risk reduction associated with high compliance with bisphosphonates do not appear to be confounded by healthy behaviors and factors associated with medication compliance except possibly for hip fracture. Further work is needed to assess the existence of a healthy adherer effect for fracture outcomes in other populations and how best to control for this potential confounder.
